# Supplementary material for: Lactobacillus rhamnosus CY12 Enhances Intestinal Barrier Function by Regulating Tight Junction Protein Expression, Oxidative Stress, and Inflammation Response in Lipopolysaccharide-Induced Caco-2 Cells
Source: Int J Mol Sci. 2022 Sep 22;23(19):11162. doi: 10.3390/ijms231911162 (PMC9569798; doi:10.3390/ijms231911162)
Supplement: Supplementary file 1 [file ijms-23-11162-s001.zip › ijms-1906750-supplementary.pdf]

International Journal of
Molecular Sciences

MDPI

Supplementary Materials

Supplementary Table S1. Sequences of primers used for quantitative real-time PCR.

Gene	Primer Sequences(5′-3′)
GAPDH	F:AGAAGGCTGGGGCTCATTTG<br>R:AGGGGCCATCCACAGTCTTC
IL-1β	F:TGAGCACCTTCTTTCCCTTC<br>R:GTCATTACTTTCTTCTCCTTGTAC
TNF-α	F:GGCAGTCAGATCATCTTCTCGAAC<br>R:TGGTAGGAGACGGCGATGC
IL-6	F:CACAGATGAAGGTGGGAAGGATG<br>R:TGCTAAGCAAACAGGCACGACTA
IL-8	F:CTGGCCGTGGCTCTCTTG<br>R:GGGTGGAAAGGTTTGGAGTATG
NF-κB	F:GGATTTCGTTTCCGTTATGTATG<br>R:TCCTTGGGTCCAGCAGTTA
TLR4	F:GCTTCTTGCTGGCTGCATAA<br>R:GAAATGGAGGCACCCCTTC
Occludin	F:GACTATGTGGAAAGAGTTGAC<br>R:ACCGCTGCTGTAACGAG
ZO-1	F:ACAACATACAGTGACGCTTC<br>R:ATTATCCATTGAAACTCCGTTAAC
Claudin	F:TGGTCAGGCTCTCTTCACTG<br>R:TTGGATAGGGCCTTGGTGTT
CAT	F:TGTTGAAGATGCGGCGAG<br>R:ATGAGAGGGTAGTCCTTGTG
SOD	F:ATCCTCTATCCAGAAAACACG<br>R:ACACCACAAGCCAAACGAC
GSH-Px	F:CAACCAGTTTGGGCATCAG<br>R:CACCGTTCACCTCGCAC
